# Supplementary material for: Environmental DNA monitoring of oncogenic viral shedding and genomic profiling of sea turtle fibropapillomatosis reveals unusual viral dynamics
Source: Commun Biol. 2021 May 12;4:565. doi: 10.1038/s42003-021-02085-2 (PMC8115626; doi:10.1038/s42003-021-02085-2)
Supplement: Supplementary file 2 — Supplementary Information [file 42003_2021_2085_MOESM2_ESM.pdf]

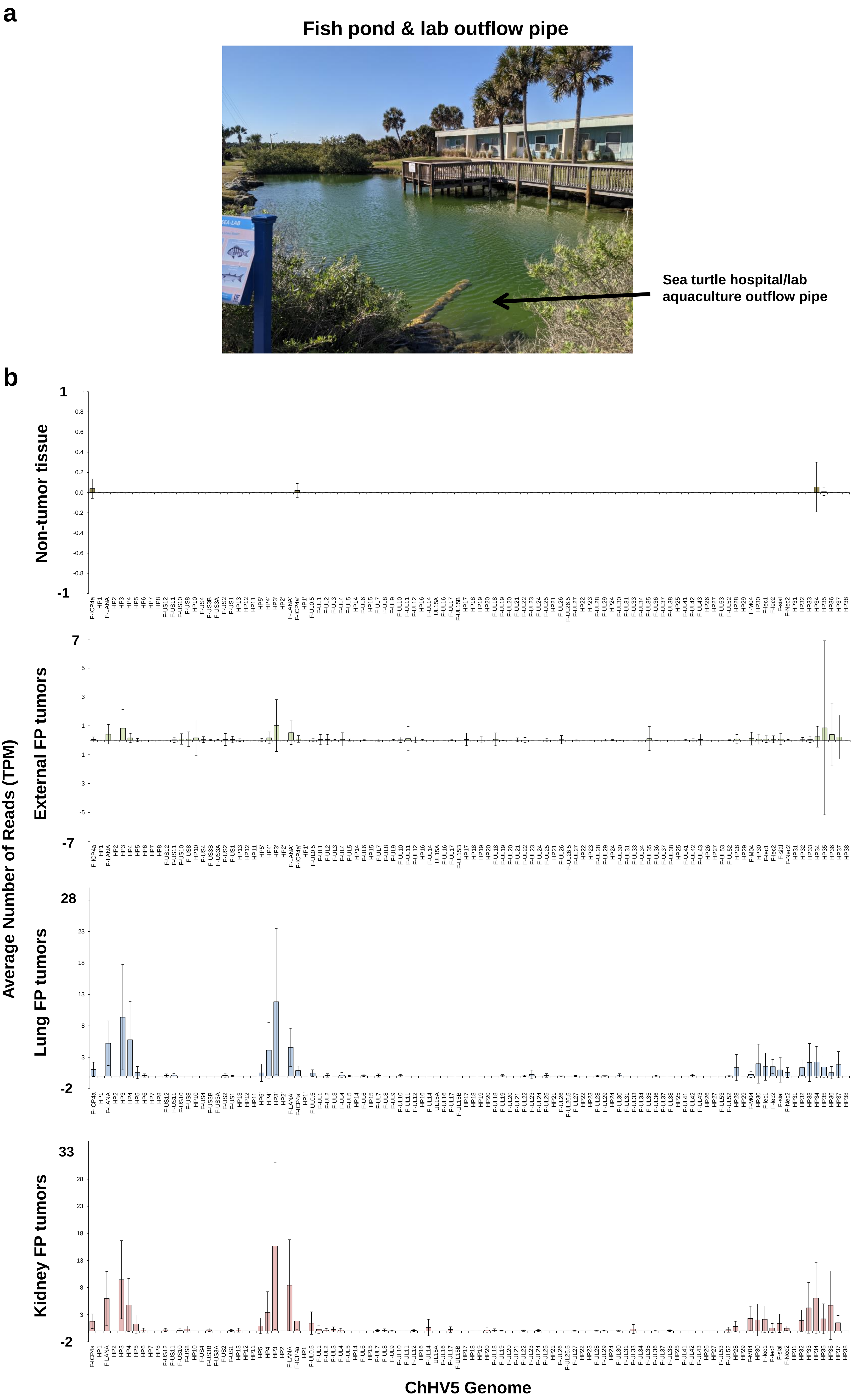

Supplementary Figure 1. Additional gene level ChHV5 transcriptomics analysis. a) Image of the fishpond at the University of Florida’s Whitney Laboratory for Marine Bioscience, in which ChHV5 was detected from eDNA water samples. Sea turtle hospital tanks and the lab’s aquaculture tanks outflow into the fishpond. **b)** Bar graph showing level of ChHV5 viral transcript expression of every gene in the ChHV5 genome, averaged by tissue type. Reads were first aligned from RNA-seq samples to the ChHV5 genome[66] via Bowtie2 and read counts generated per viral gene using htseq-count. Reads were normalized by gene length and sequencing depth to transcripts per million (TPM) and averaged across samples for the following groups: non-tumor tissue n = 20, external fibropapillomatosis tumors n = 56, lung fibropapillomatosis tumors n = 7, and kidney fibropapillomatosis tumors n = 6.

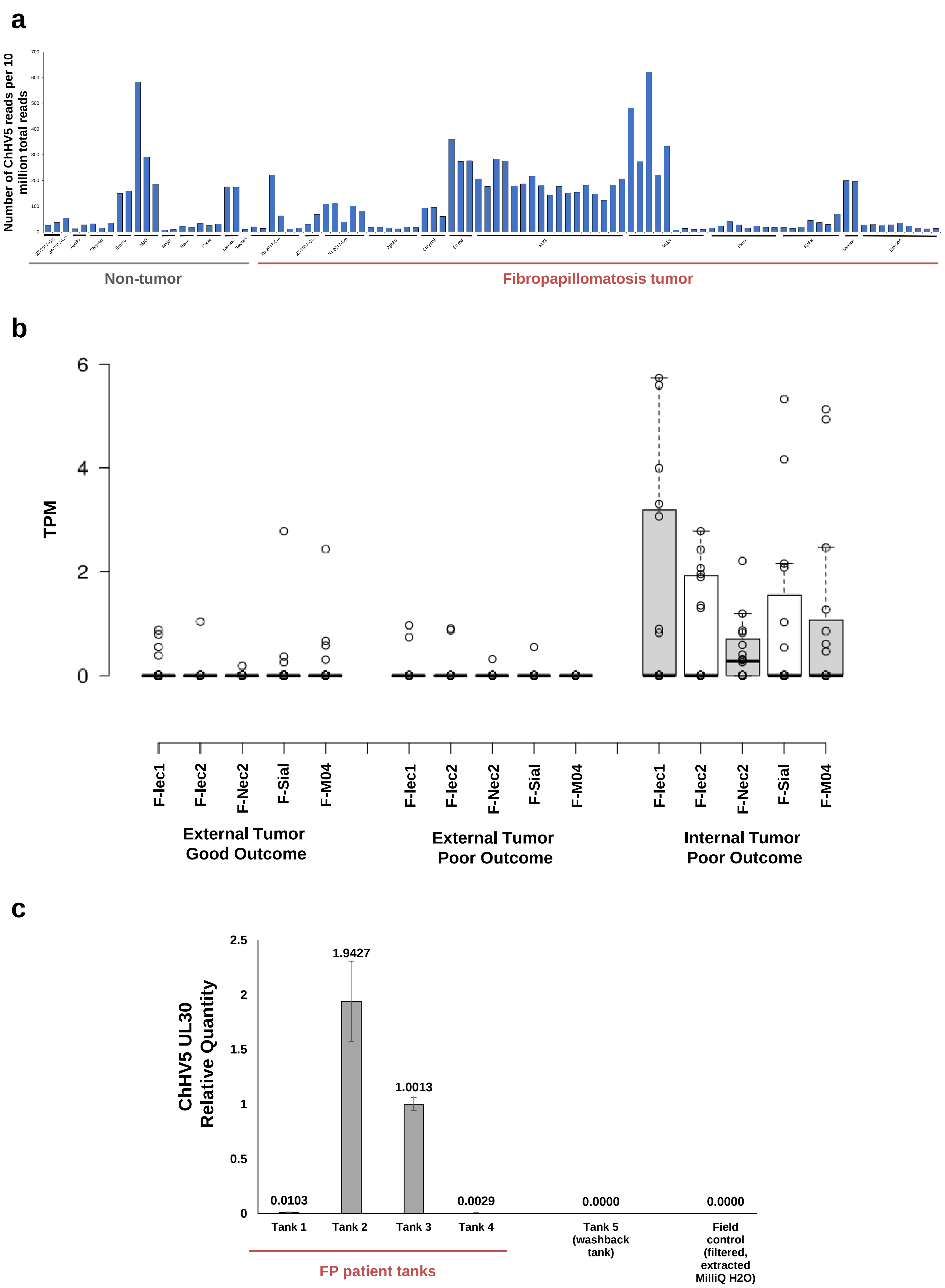

Supplementary Figure 2. ChHV5 transcript levels compared with rehabilitation outcome. a) Bar graph showing total level of ChHV5 RNA among samples by individual patient, as assessed by RNA-seq. Reads were normalized by sequencing depth to number of viral reads per 10 million total reads per sample. Bars show individual samples per patient. **b)** ChHV5 F-lec1, F-lec2, F-Nec, F-Sial and F-M04 gene expression box plot with Tukey whiskers, by patient outcome and tumor location, as detected by RNA-seq. Individual samples for each gene and sample type are shown by the open points. **c)** Detection and quantification of ChHV5 UL30 gene DNA in patient tank water by qPCR. Samples from tanks 1-5 are the five constituent samples which were pooled (an aliquot of each) for WGS (Fig. 5a). Tanks 1-4 housed FP-afflicted patients, while tank 5 housed FP-free post-hatchling washbacks. Amplification ratios for tanks 1-3 were 1.0, for tank 4 was 0.3, and for tank 5 and the negative field control were 0.0. Error bars denote standard deviation of three technical replicates.

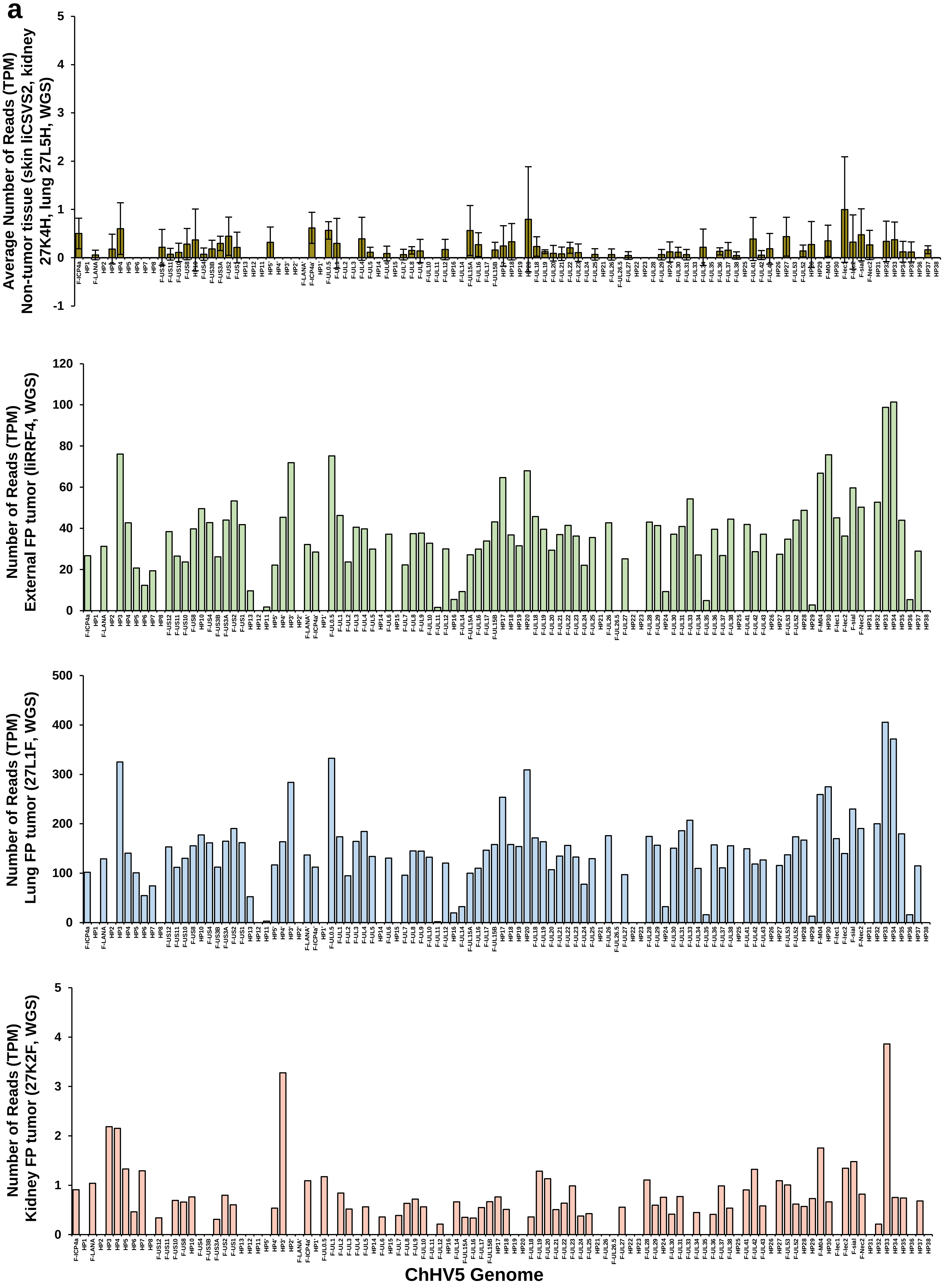

Supplementary Figure 3. Additional gene level ChHV5 genomic (DNA-seq) analysis. a) Bar graph showing level of ChHV5 viral DNA reads of every protein coding gene in the ChHV5 genome. For the three non-tumor samples reads per gene were averaged. Reads were first aligned from DNA-seq samples to the ChHV5 genome[66] via Bowtie2 and read counts generated using htseq-count. Reads were normalized by gene length and sequencing depth to transcripts per million (TPM). Per sample type: non-tumor tissue n = 3, external FP tumor n = 1, lung FP tumor n = 1, and kidney FP tumor n = 1.

a

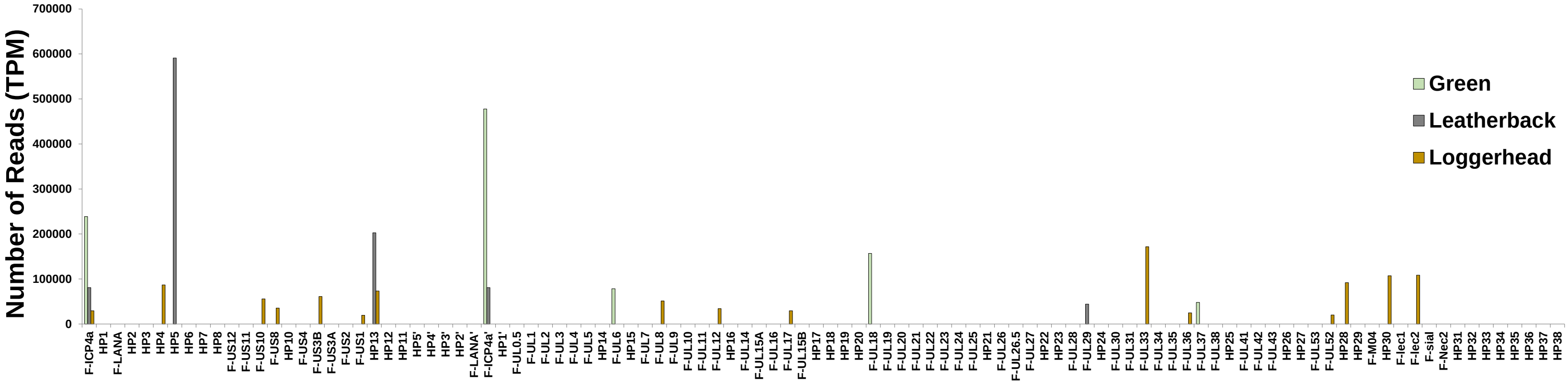

b

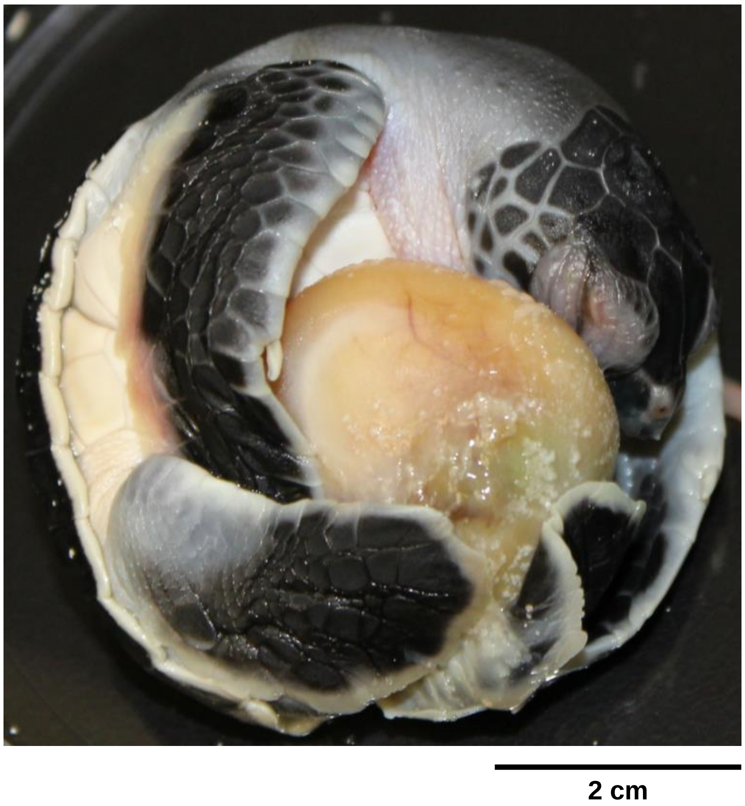

Supplementary Figure 4. Additional hatchling ChHV5 genomic (DNA-seq) analysis. a) Bar graph showing level of ChHV5 viral DNA reads of every coding gene in the ChHV5 genome, for each of the three hatchling species sequenced (WGS): green (*C. mydas)*, loggerhead (*C. carretta*), and leatherback (*D. coriacea*). Reads were first aligned from DNA-seq samples to the ChHV5 genome[66] via Bowtie2 and read counts generated using htseq-count. Reads were normalized by gene length and sequencing depth to transcripts per million (TPM). **b)** Image of the deceased unhatched green sea turtle in whose tissue ChHV5 was detected by qPCR at low levels.

Supplementary Table 1. Result of eDNA-based detection of ChHV5 in the institutional fishpond, which receives filtered outflow seawater from the aquaculture and sea turtle patient tanks (Supplementary Fig. 1a), and in pooled and individual leech samples (Fig. 1e), and sand eDNA samples (Fig. 2b). The table reports the amplification ratio for each sample and the number of qPCR wells that ChHV5 was positively detected in, out of how many technical replicate wells were run. For low input samples such as field eDNA, amplification ratio (the proportion of positive amplification detection relative to attempted reactions) is a commonly utilized measure[110].

**Sample**	**Amplification ratio**	**Positive detection / Number of technical replicates score**
Water samples:		
Fishpond Biological Replicate A	0.5	3/6
Fishpond Biological Replicate B	1.0	6/6
Fishpond Biological Replicate C	1.0	6/6
Field negative control	0.0	0/6
Pooled leech samples:		
07-2015-Cm Tumor	1.0	3/3
09-2015-Cc Non-tumor (FP-free loggerhead)	0.0	0/3
36-2020-Cm “Richard Dawkins” Tumor 1	1.0	6/6
36-2020-Cm “Richard Dawkins” Tumor 2	0.8	5/6
36-2020-Cm “Richard Dawkins” Non-tumor 1	0.0	0/6
36-2020-Cm “Richard Dawkins” Non-tumor 2	0.0	0/6
52-2020-Cm “Bruno Hofer” Tumor 1	0.5	3/6
52-2020-Cm “Bruno Hofer” Tumor 2	1.0	6/6
52-2020-Cm “Bruno Hofer” Non-tumor 1	1.0	6/6
52-2020-Cm “Bruno Hofer” Non-tumor 2	1.0	6/6
78-2020-Cm “Ruth Gates” Tumor 1	0.7	4/6
78-2020-Cm “Ruth Gates” Tumor 2	0.5	3/6
78-2020-Cm “Ruth Gates” Non-tumor 1	0.0	0/6
78-2020-Cm “Ruth Gates” Non-tumor 2	1.0	6/6
Individual leech samples:		
Leech 1 (02-2021-Cm “Broccoli”)	1.0	6/6
Leech 2 (02-2021-Cm “Broccoli”)	0.2	1/6
Leech 3 (02-2021-Cm “Broccoli”)	0.8	5/6
Leech 4 (02-2021-Cm “Broccoli”)	0.3	2/6
Leech 5 (02-2021-Cm “Broccoli”)	0.8	5/6
Leech 6 (02-2021-Cm “Broccoli”)	1.0	6/6
Leech 7 (02-2021-Cm “Broccoli”)	1.0	6/6
Leech 8 (02-2021-Cm “Broccoli”)	0.2	1/6
Leech 9 (02-2021-Cm “Broccoli”)	0.8	5/6
Leech 10 (02-2021-Cm “Broccoli”)	0.8	5/6

Leech 11 (02-2021-Cm “Broccoli”)	0.5	3/6
Leech 12 (02-2021-Cm “Broccoli”)	0.3	2/6
Leech 13 (02-2021-Cm “Broccoli”)	0.8	5/6
Leech 14 (02-2021-Cm “Broccoli”)	1.0	6/6
Leech 15 (02-2021-Cm “Broccoli”)	0.7	4/6
Leech 16 (02-2021-Cm “Broccoli”)	0.3	2/6
Leech 17 (02-2021-Cm “Broccoli”)	1.0	6/6
Leech 18 (02-2021-Cm “Broccoli”)	0.0	0/6
Leech 19 (02-2021-Cm “Broccoli”)	0.8	5/6
Leech 20 (02-2021-Cm “Broccoli”)	0.8	5/6
Leech 21 (02-2021-Cm “Broccoli”)	0.3	2/6
Leech 22 (02-2021-Cm “Broccoli”)	1.0	6/6
Leech 23 (02-2021-Cm “Broccoli”)	1.0	6/6
Leech 24 (02-2021-Cm “Broccoli”)	1.0	6/6
Leech 25 (02-2021-Cm “Broccoli”)	0.3	2/6
Leech 26 (02-2021-Cm “Broccoli”)	0.0	0/6
Leech 27 (02-2021-Cm “Broccoli”)	0.0	0/6
Leech 28 (02-2021-Cm “Broccoli”)	0.2	1/6
Leech 29 (02-2021-Cm “Broccoli”)	0.2	1/6
Leech 30 (02-2021-Cm “Broccoli”)	0.2	1/6
Sand samples (“Archie Carr” 49-2020-Cm):		
ac_17th Sep. Biological Replicate A	0.3	2/6
ac_17th Sep. Biological Replicate B	1.0	6/6
ac_17th Sep. Biological Replicate C	1.0	6/6
ac_14th Oct. Biological Replicate A	1.0	6/6
ac_14th Oct. Biological Replicate B	1.0	6/6

Supplementary Table 2. Fibropapillomatosis histology, inclusion body presence or absence as identified by hematoxylin and eosin (H&E) staining. For the stromal cellularity score and the lymphocytic inflammation score, ranks are from 0 to 3, with 0 being the least and 3 being the most.

Tumor	Inclusion bodies	Cellularity score	Lymphocytic inflammation score	Ulceration / heterophilic inflammation
1. New Growth External	No	2	2	No
2. New Growth External	No	2	0	Yes
3. New Growth External	No	1	0	Yes
4. New Growth External	No	2	1	Yes
5. New Growth External	No	2	2	No
6. New Growth External	No	2	2	No
7. New Growth External	No	1	1	Yes
8. New Growth External	No	2	0	Yes
9. New Growth External	No	1	0	Yes
10. Established External	No	3	0	No
11. Established External	No	1	0	Yes
12. Established External	No	1	0	No
13. Established External	No	1	0	Yes
14. Established External	No	1	0	No
15. Regrowth External	No	3	1	No
16. Established External (basal)	No	1	1	No
17. Eye	No	3	0	No
18. Internal Kidney	No	2	0	No
19. Internal Kidney	No	2	0	No

Supplementary Table 3 PV1 transcriptomics analysis. Reads were aligned from DNA-seq samples to the *Chelonia mydas* (Cm) PV1 genome (https://www.ncbi.nlm.nih.gov/nuccore/EU493091?report=genbank) and the *Caretta caretta* (Cc) PV1 genome (https://www.ncbi.nlm.nih.gov/nuccore/EU493092?report=genbank) via bowtie2. Dc, *Dermochelys coriacea*.

Sample	Individual ID	Source	Cm PV1 reads detected	Cc PV1 reads detected
HiSeq 3000:				
27K2F	27-2017-Cm	Kidney FP tumor	0	1 *(Note: singleton, no mate pair. 0.037 RPTM)*
27K4H	27-2017-Cm	Kidney non-tumor	0	0
27L1F	27-2017-Cm	Lung FP tumor	0	0
27L5H	27-2017-Cm	Lung non-tumor	0	0
liCSVS2dna	Lilac	Skin punch non-tumor	0	0
liRRF4dna	Lilac	New growth external FP tumor	0	0
GreenHatch DNA	Cm hatchling	Hatchling flipper cross-section (non-tumor)	0	0
LoggerHatch DNA	Cc hatchling	Hatchling flipper cross-section (non-tumor)	0	0
LeatherHatch DNA	Dc hatchling	Hatchling flipper cross-section (non-tumor)	0	0
25AugPl	Tangled	Plasma	0	0
17MayPl	Remi	Plasma	0	0
17SepPl	Remi	Plasma	0	0
24JunPl	Rollie	Plasma	0	0
24AugPl	Rollie	Plasma	0	0
23MayPl	RT	Plasma	0	0
23AugPl	RT	Plasma	0	0
02JanPl	Chrystal	Plasma	0	0
02AugPl	Chrystal	Plasma	0	0
34SeptPl	34-2017-Cm	Plasma	0	0
Tank eDNA	Mix	eDNA from tank water	0	0
NovaSeq 6000:				
27L1Fdna (same DNA sample as above)	27-2017-Cm	Lung FP tumor	0	0
27L5Hdna (same DNA sample as above)	27-2017-Cm	Lung non-tumor	0	0
flSCBLdna	Flower	Whole Blood	0	0
flSCEYFdna	Flower	External FP tumor	0	0
flSCINFdna	Flower	External FP tumor	0	0
poSCBLdna	Poppy	Whole Blood	0	0

poSCTFdna	Poppy	External FP tumor	0	0
yuSB1Hdna	Yucca	Skin punch non-tumor	0	0
yuLKHdna	Yucca	Kidney non-tumor	0	0
yuLIRSFdna	Yucca	External FP tumor	0	0
yuRIRSFdna	Yucca	External FP tumor	0	0
yuTSFdna	Yucca	External FP tumor	0	0
yuRERFdna	Yucca	External FP tumor	0	0
yuRKTGFdna	Yucca	Kidney FP tumor	0	0
yuRKTMFdna	Yucca	Kidney FP tumor	0	1 (*Note: singleton, no mate pair. 0.014 RPTM*)
yuRKTW1Fdna	Yucca	Kidney FP tumor	0	0
TABT-Cm	Aladar	Bladder FP tumor	0	0
TABH-Cm	Aladar	Bladder non-tumor	0	0

Supplementary Table 4. *Chelonia mydas* 16S rRNA and ChHV5 UL30 primer, probe and synthetic gene fragment sequences for qPCR. Both turtle and ChHV5 probes have 5’ FAM dye and an Iowa black 3’ quencher and an internal ZEN quencher. Primers, probes and gene fragments were synthesized by Integrated DNA Technologies, IDT.

Name	Sequence
Green Turtle (Atlantic) 16S rRNA Fwd	TGC AAA AGC GGG AAT AAC AC
Green Turtle (Atlantic) 16S rRNA Rev	TCG CCC CAA CCA AAA ATA TAG
Green Turtle (Atlantic) 16S rRNA Probe	CAA CTA TCT ATA CCC ACT CAC TCT AAG GAC CTA TAA
Green Turtle (Atlantic) 16S rRNA Synthetic Gene Fragment	GCC TCT AGC AAC ACA CAA GTA TTG GAG GTA ATG CCT GCC CAG TGA CAT TGT TAA ACG GCC GCG GTA TCC TAA CCG TGC AAA GGT AGC GTA ATC ACT TGT CTT TTA AAT AAA GAC TAG AAT GAA TGG CCA AAC GAG GTT CTA CCT GTC TCT TAC AAA CAA TCA GTG AAA TTG ATC TCC CCG TGC AAA AGC GGG AAT AAC ACT ATA AGA CGA GAA GAC CCT GTG GAA CTT TAA ATA CAG ATC AAC TAT CTA TAC CCA CTC ACT CTA AGG ACC TAT AAC TAA CTA GTA CTT GAC CTA TAT TTT TGG TTG GGG CGA CCT CGG AGT AAA ACA AAA CCT CCG AAA AAA GAA TAC ACT TCT TAA CCT AGA CCC ACA ATT CAA AGT GCC AAC GGC AAA ATG ATC CAA TAT ATT TGA TCA ACG AAC CAA GCT ACC CCA GGG ATA ACA GCG CAA TCC CAT CCT AGA GTT CCT ATC GAC GAT GGG GTT TAC GAC CTC GAT GTT GGA TCA G
ChHV5 UL30 Fwd[38]	AACGCTTGCTTTTGGACAAG
ChHV5 UL30 Rev[38]	CCAGCGGGTGTGAATAAAAT
ChHV5 UL30 Probe[38]	TGGCCATCAAGCTGACGTGCA
ChHV5 UL30 Synthetic Gene Fragment	CTC AAG GAG CTA AAA GCC GGC GAA GAT TAC GAG GAG TTC AAA GTT CAG GGC ATG TCC CTT TAC TAC GTC AAG CCT CAC GTG CGC CGC AGC CTT TTG GGC GAG CTG CTG ACC GAC TGG CTG GCA CTC AGG AAA AAA ATC CGC GCA TCG ATG AAA ACC GCA CCG AGC GAC CAA CGC TTG CTT TTG GAC AAG CAG CAG CTG GCC ATC AAG CTG ACG TGC AAT TCC GTT TAC GGG TTT ACC GGC GTG GCC ACC GGA TTC CTA CCT TGT CTG GAG GTG GCG GCC ACG GTC ACC ACC GTA GGG CGC GAC ATG CTT CTG GCC ACG CGG GAT TTT ATT CAC ACC CGC TGG GGA ACG GAT TTC GAA GCC TTG TTG GTC GAC GCG CCG GAA CTG GCC GCG TTT CGA CGA CCC GAA TCG CTT TTT GGC CTG CGT GTC ATT TAC GGG GAC ACC GAC TCG GTG TTC GTG CTG TGC ACC GGC GTC GCA GCA GAG

Supplementary References
(numbered in accordance with reference number in the main manuscript)

66 Ackermann, M. et al. The Genome of Chelonid Herpesvirus 5 Harbors Atypical Genes. *PLoS ONE* **7**, e46623, doi:10.1371/journal.pone.0046623 (2012).

110 Shamblin, B. M. et al. Loggerhead turtle eggshells as a source of maternal nuclear genomic DNA for population genetic studies. Molecular Ecology Resources 11, 110-115, doi:https://doi.org/10.1111/j.1755-0998.2010.02910.x (2011).
