# Supplementary material for: Environmental DNA monitoring of oncogenic viral shedding and genomic profiling of sea turtle fibropapillomatosis reveals unusual viral dynamics
Source: Commun Biol. 2021 May 12;4:565. doi: 10.1038/s42003-021-02085-2 (PMC8115626; doi:10.1038/s42003-021-02085-2)
Supplement: Supplementary file 3 — Descriptions of Additional Supplementary Files [file 42003_2021_2085_MOESM3_ESM.pdf]

Descriptions of Additional Supplementary Files

Supplementary Data 1
Description: Detailed information on each sample sequenced as part of this study.

Supplementary Data 2
Description: Count table of ChHV5 gene expression in each RNA-seq sample. Expression level of every ChHV5 gene is provided in TPM.

Supplementary Data 3
Description: Sample information for each sample in which ChHV5 was assessed by UL30 qPCR (Fig. 5c).

Supplementary Data 4
Description: Tumor surface area and ChHV5 concentration data used for the correlation reported in Fig. 2f.
