# Supplementary material for: Environmental DNA monitoring of oncogenic viral shedding and genomic profiling of sea turtle fibropapillomatosis reveals unusual viral dynamics
Source: Commun Biol. 2021 May 12;4:565. doi: 10.1038/s42003-021-02085-2 (PMC8115626; doi:10.1038/s42003-021-02085-2)
Supplement: Supplementary file 8 — Reporting Summary [file 42003_2021_2085_MOESM8_ESM.pdf]

nature research

Corresponding author(s): David Duffy

Last updated by author(s): Feb 17, 2021

Reporting Summary

Nature Research wishes to improve the reproducibility of the work that we publish. This form provides structure for consistency and transparency in reporting. For further information on Nature Research policies, see our Editorial Policies and the Editorial Policy Checklist.

Statistics

For all statistical analyses, confirm that the following items are present in the figure legend, table legend, main text, or Methods section.

n/a	Confirmed	
[ ]	[x]	The exact sample size (*n*) for each experimental group/condition, given as a discrete number and unit of measurement
[ ]	[x]	A statement on whether measurements were taken from distinct samples or whether the same sample was measured repeatedly
[ ]	[x]	The statistical test(s) used AND whether they are one- or two-sided<br>*Only common tests should be described solely by name; describe more complex techniques in the Methods section.*
[x]	[ ]	A description of all covariates tested
[ ]	[x]	A description of any assumptions or corrections, such as tests of normality and adjustment for multiple comparisons
[ ]	[x]	A full description of the statistical parameters including central tendency (e.g. means) or other basic estimates (e.g. regression coefficient) AND variation (e.g. standard deviation) or associated estimates of uncertainty (e.g. confidence intervals)
[x]	[ ]	For null hypothesis testing, the test statistic (e.g. *F*, *t*, *r*) with confidence intervals, effect sizes, degrees of freedom and *P* value noted<br>*Give P values as exact values whenever suitable.*
[x]	[ ]	For Bayesian analysis, information on the choice of priors and Markov chain Monte Carlo settings
[x]	[ ]	For hierarchical and complex designs, identification of the appropriate level for tests and full reporting of outcomes
[x]	[ ]	Estimates of effect sizes (e.g. Cohen's *d*, Pearson's *r*), indicating how they were calculated

Our web collection on statistics for biologists contains articles on many of the points above.

Software and code

Policy information about availability of computer code

Data collection	No data was collected through computer code for this study.
Data analysis	Trim Galore (version 0.6.5)<br>FastQC (version 0.11.9)<br>BioVenn (version 2007-2020)<br>Degust (2020)<br>PANNZER2 (version 2)<br>BLASTp (2019)<br>htseq count (version 0.6.1p1)

For manuscripts utilizing custom algorithms or software that are central to the research but not yet described in published literature, software must be made available to editors and reviewers. We strongly encourage code deposition in a community repository (e.g. GitHub). See the Nature Research guidelines for submitting code & software for further information.

Data

Policy information about availability of data

All manuscripts must include a data availability statement. This statement should provide the following information, where applicable:

- Accession codes, unique identifiers, or web links for publicly available datasets
- A list of figures that have associated raw data
- A description of any restrictions on data availability

The sequencing data including raw reads are deposited in NCBI (https://www.ncbi.nlm.nih.gov/) under BioProject ID: PRJNA449022 (https://www.ncbi.nlm.nih.gov/bioproject/PRJNA449022).

Field-specific reporting

Please select the one below that is the best fit for your research. If you are not sure, read the appropriate sections before making your selection.

- [x] Life sciences
- [ ] Behavioural & social sciences
- [ ] Ecological, evolutionary & environmental sciences

For a reference copy of the document with all sections, see nature.com/documents/nr-reporting-summary-flat.pdf

Life sciences study design

All studies must disclose on these points even when the disclosure is negative.

Sample size	Sample size calculations were not performed. Sample sizes were selected based on our previously published work, which provided information on the samples size required for RNA-seq and qPCR to provide statistically significant results. Please note also that given the nature of the sampling, opportunistic sampling at a rehabilitation hospital for endangered sea turtles (i.e. not laboratory model animals), availability of certain tumor types was opportunistic.
Data exclusions	A single RNA-seq sample was excluded from the analysis as the reads did not pass QC criteria.
Replication	Independent samples from independent tumors and individuals were used. While only applicable to the established external tumor cohort, RNA-seq results generally replicated earlier findings from two smaller external tumor cohorts (Duffy et al. Comms Bio 2018; Blackburn et al. BioRxiv 2020 https://www.biorxiv.org/content/10.1101/2020.10.29.360834v1).
Randomization	Please note also that interventions to groups of patients were not conducted as part of this study, minimising the need for randomisation. Turtles to be included in the study were naturally randomly selected, we had no input into which FP afflicted turtles stranded and were admitted to our hospital, similarly we had no influence over rehabilitation process or surgical decisions, or decisions to euthanize patients (euthanized turtles provided access for internal sampling). Therefore, all patients included in the study were randomly included by processes we did not control (natural stranding events, state level assignment of turtles to individual rehab hospitals and independent veterinary decisions), with our sampling activities being opportunistic as the opportunities arose.
Blinding	Blinding was not relevant to this study. Groups were not artificially generated by researchers, either a tumor was internal or external, and either an animal was released or died in rehab. For the transcriptomics analyses robust unbiased analysis types were used to investigate differences between tumor groups (e.g. PCA plots and DE analysis).

Reporting for specific materials, systems and methods

We require information from authors about some types of materials, experimental systems and methods used in many studies. Here, indicate whether each material, system or method listed is relevant to your study. If you are not sure if a list item applies to your research, read the appropriate section before selecting a response.

Materials & experimental systems

n/a	Involved in the study	
x		Antibodies
x		Eukaryotic cell lines
x		Palaeontology and archaeology
	x	Animals and other organisms
x		Human research participants
x		Clinical data
x		Dual use research of concern

Methods

n/a	Involved in the study	
x		ChIP-seq
x		Flow cytometry
x		MRI-based neuroimaging

Animals and other organisms

Policy information about studies involving animals; ARRIVE guidelines recommended for reporting animal research

Laboratory animals	N/A
Wild animals	N/A No wild animals were caught, transported or killed as part of this research. We obtained samples from animals undergoing rehabilitation. All husbandry, veterinary care, transport and release of these animals was conducted by the relevant licensed authorities (the sea turtle stranding network and the hospitals conducted such activities under their state permits and the supervision of the Florida Fish and Wildlife Conservation Commission and the U.S. Fish and Wildlife Service), not by the researchers. The researchers had no role in any of these aspects of animal care, being permitted to receive samples, not to care or house the animals.
Field-collected samples	N/A
Ethics oversight	Ethical oversight: IACUC committee of the University of Florida.<br>Endangered species permitting: the Florida Fish and Wildlife Conservation Commission.

Note that full information on the approval of the study protocol must also be provided in the manuscript.
